# Impact of WHO 2010 Guidelines on Antiretroviral Therapy Initiation among Patients with HIV-Associated Tuberculosis in Clinics with and without Onsite HIV Services in the Democratic Republic of Congo

**DOI:** 10.1155/2016/1027570

**Published:** 2016-08-09

**Authors:** Marcel Yotebieng, Martine Tabala, Marie Louise Batumbula, Landry Wenzi, Emmanuel Basaki, Eugenie Mungoyo, Richard Mangala, Frieda Behets

**Affiliations:** ^1^Division of Epidemiology, College of Public Health, The Ohio State University, 304 Cunz Hall, 1841 Neil Avenue, Columbus, OH 43210-1351, USA; ^2^Department of Epidemiology, The University of North Carolina at Chapel Hill, 135 Dauer Drive, 2101 McGavran-Greenberg Hall, CB No. 7435, Chapel Hill, NC 27599-7435, USA; ^3^School of Public Health, The University of Kinshasa, Kinshasa, Congo; ^4^School of Medicine, The University of North Carolina at Chapel Hill, 333 South Columbia Street, MacNider Hall, Room No. 348, CB No. 7240, Chapel Hill, NC 27599-7240, USA

## Abstract

*Background*. We assessed the impact of WHO's 2010 guidelines that removed the requirement of CD4 count before ART, on timely initiation of ART among HIV/TB patients in the Democratic Republic of Congo (DRC).* Methods*. Data collected to monitor implementation of provider initiated HIV testing and counseling (PITC) and linkage to HIV care from 65 and 13 TB clinics in Kinshasa and Kisangani, respectively, between November 2010 and June 2013.* Results*. Prior to the WHO's 2010 guidelines, in Kinshasa, 79.1% (401/507) of HIV/TB patients referred for HIV services were initiated on ART in clinics with onsite ART services compared to 50.0% (63/123) in clinics without. Following the implementation of the new guidelines, 89.8% (714/795) and 93.0% (345/371) of HIV/TB patients referred for HIV services were initiated on ART, respectively, in clinics with onsite and without onsite ART services. Similarly, in Kisangani, 69.7% (53/120) and 36.4% (16/44) in clinics with and without onsite ART service, respectively, were initiated on ART prior to the 2010 guidelines and 88.8% (135/152) and 72.6% (106/146), respectively, after the new guidelines.* Conclusion*. Though implementation of the 2010 guidelines increased the proportion of HIV/TB patients initiated on ART substantially, it remained below the 100% target, particularly in clinics without onsite ART services.

## 1. Introduction

Tuberculosis (TB) is the main cause of morbidity and mortality among human immunodeficiency virus (HIV) infected persons worldwide [1, 2]. According to the latest World Health Organization (WHO) Global TB report [3], TB accounted for a third of all deaths among people living with HIV/AIDS (PLWH) in 2014. In addition to appropriate anti-TB treatment, administration of trimethoprim-sulphamethoxazole (cotrimoxazole) prophylaxis (CPT) and early initiation of antiretroviral therapy (ART) are critical to improving outcomes in HIV-positive TB (HIV/TB) patients [4–7]. Yet, though coverage of CPT among TB patients known to be coinfected with HIV reached 87% in the African region in 2013, that of ART remained low at 69% [3].

The Democratic Republic of Congo (DRC) carries the 9th highest burden of TB worldwide and 14% of patients with TB in DRC are also infected with HIV. Coverage of CPT and ART among HIV/TB patients in DRC substantially lags behind the regional and international averages though 70% and 48% of them were initiated on CPT and ART, respectively, in 2013, compared to 24% and 6% in 2010 [3]. Possible explanations for this low performance include the lack of integration of HIV services into TB care and poor access to CD4 count prior to the implementation of the WHO 2010 guidelines. In the DRC, TB treatment services are highly decentralized to the level of primary health care service while HIV treatment services are generally available only at the level of district clinics [8, 9]. Implementation data showed that the more integrated the HIV services into TB clinical settings, the higher the uptake among TB patients [10, 11]. Moreover, prior to the adoption of the 2010 WHO guidelines [12] which recommended that ART should be given to all patients regardless of CD4 cell count starting as soon as possible after TB treatment is tolerated and not later than within 8 weeks [13], all non-Stage 4 TB patients needed a CD4 count measurement to assess their eligibility to ART as per 2006 guidelines [13]. Yet, CD4 count evaluation was only available at the provincial laboratory and patients physically had to present at the laboratory to get their blood sample taken. How removal of the CD4 count requirement before ART has impacted access to ART for HIV/TB patients and the remaining role of centralized HIV services in the low coverage has not yet been assessed.

The aims of this study were to assess the impact of the WHO 2010 guidelines and the remaining role of centralized HIV services on ART initiation among HIV/TB patients in the Democratic Republic of Congo (DRC).

## 2. Methods

### 2.1. Setting and Participants

In 2010, the Schools of Public Health of the University of North Carolina at Chapel Hill (UNC) and of the University of Kinshasa (KSPH) with funding from the President's Emergency Plan For AIDS Relief (PEPFAR) through the US Centers for Disease Control and Prevention (CDC) and in collaboration with the DRC's National TB Program (PNLT) and the National AIDS Control Program (PNLS) started providing operational and logistical support to TB clinics in Kinshasa and in Kisangani, the capital of the Oriental Province, for the implementation of HIV/TB collaborative activities. These included provider initiated counseling and testing for all patients seeking care in the clinics with symptoms suggestive of TB, provision of CPT, and linkage to HIV services for HIV/TB infected patients.

In addition to anti-TB medications, all TB patients diagnosed with HIV were initiated on CPT immediately. As of January 2012 in Kisangani and March 2012 in Kinshasa, the supported TB clinics started implementing the 2010 WHO ART initiation recommendations instead of the 2006 guidelines [13]. In line with the 2006 WHO guidelines, HIV/TB patients were referred to HIV clinics after at least two weeks and within eight weeks of anti-TB treatment initiation for evaluation of their eligibility for ART. Patients in clinical stage IV were immediately eligible for ART and the remaining were sent to the central PNLS laboratory in each city for CD4 count measurement. All those with CD4 count < 200 cells/mm^3^ were eligible to start ART. A referral and counter referral form was used to exchange key information between the HIV clinics and TB clinics including information on ART initiation (date and regimen). This information was recorded in the integrated TB and HIV registry at the TB clinic. At the end of TB treatment, follow-up in TB clinics was ended and HIV/TB patients were referred to HIV clinics.

### 2.2. Data Source and Quality Control

For monitoring purposes, at the end of each month, the TB nurse (in charge of TB treatment and HIV/TB registry) used information from the integrated TB and HIV registry in each clinic to report the number of new TB patients diagnosed during the month, the number of those with known HIV status prior to TB diagnosis, the number of those with unknown HIV status counseled and tested for HIV, the number of HIV/TB coinfected patients, the number of coinfected patients initiated on CPT, the number of coinfected patients with at least two weeks of anti-TB treatment that were referred for HIV care and treatment, and the number of those referred who were known (returned the counter referral form) to have been initiated on ART. The aggregated data was verified and validated each month by a member of UNC/KSPH technical team during their monthly supervision visit to each clinic. Validation consisted of verifying the reports to make sure there was no double counting and getting updated information on referral and uptake of referral for patients that remained active in the clinic. The validated report was taken back to the central monitoring and evaluation team and entered into an electronic database that was used for this analysis.

### 2.3. Variable and Definitions

The main indicator of interest in this analysis was the proportion of HIV/TB patients initiated on ART before the end of their TB treatment. This proportion was calculated as the number of HIV/TB coinfected patients reported to have been initiated on ART divided by the total number of HIV/TB patients referred for HIV care and treatment. We also calculated and reported the proportion of HIV/TB coinfected patients initiated on CPT as the number of HIV/TB patients initiated on CPT divided by the total number of HIV/TB coinfected patients registered.

Another key variable that was used in this analysis as a measure of decentralization of HIV services was the integration of HIV and TB services in each clinic. TB clinics were classified in two groups based on presence or absence of onsite (located in the same health facility) HIV clinic with ART services.

Two periods were considered in the analysis: the period before and after implementation initiation of 2010 WHO guidelines, that is, before and after January 2012 in Kisangani and March 2012 in Kinshasa.

### 2.4. Data Analysis

A Log-binomial regression model was used to calculate the proportion ratios (PR) and their 95% confidence intervals (95% CI) as a measure of impact of onsite ART services and that of implementation of 2010 WHO guidelines on the proportions of patients initiated on ART. Separate analyses were done for Kinshasa and Kisangani. All analyses were performed using SAS 9.3 (Cary, NC) and all tests were performed at a 0.05 significance level.

The routine data collection was approved by the University of North Carolina at Chapel Hill Institutional Review Board and the Kinshasa School of Public Health Ethical Committee.

## 3. Results

### 3.1. Participants

Between November 2010 and June 2013, 20,912 TB cases were reported from 65 TB clinics in Kinshasa (Table 1). Clinics were added in waves of 20, 12, and 33 in the fourth quarter of 2010 (Q4 2010), Q4 2011, and Q3 2012, respectively. Thirty of those clinics had onsite ART services. Of the reported TB cases, HIV status was known in 507 (2.5%) at the time of TB diagnosis; 19,341 were counseled and tested for HIV; and 2,181 (11.3%) tested positive. Of the 2,675 TB patients known to be HIV positive during the reporting period, 2,606 (97.0%) were initiated on CPT; 1,799 (83.0%) were referred for HIV services; and 84.4% (*n* = 1523) of those referred were known to have been initiated on ART.

In Kisangani, 2,988 TB cases were reported between April 2011 and June 2013 in 13 TB clinics. The clinics were added in waves of three, three, and seven in the Q2 2011, Q3 2011, and Q3 2012, respectively (Table 1). Six of those clinics had onsite ART services. Of the reported TB cases, 175 (5.9%) were known to be HIV positive at the time of TB diagnosis. Of the 2,626 with unknown HIV status who were counseled and tested for HIV during the period, 566 (14.9%) tested positive. Of the 741 patients with HIV-associated TB, 630 (85.0%) were reported to be on CPT; 418 had documented referral for HIV care and treatment including 310 (74.2%) who were initiated on ART.

### 3.2. Impact of Implementation of 2010 WHO Guidelines and of Onsite ART Services on Proportion of HIV/TB Patients Initiated on ART

**Figure 1:**
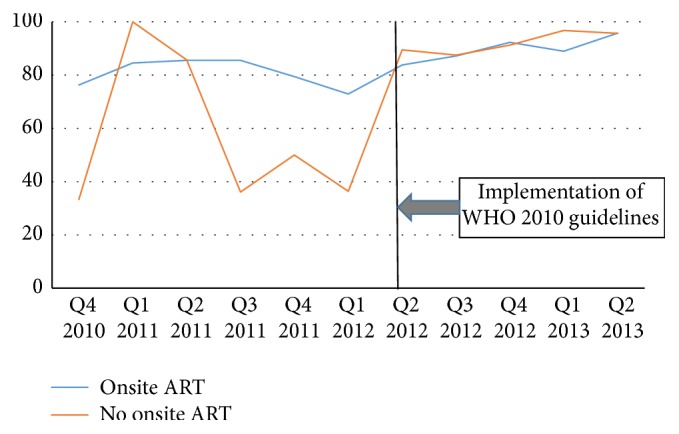
Proportions of HIV/TB coinfected patients initiated on ART by the end of TB treatment in clinics with and without onsite HIV services in Kinshasa.

**Figure 2:**
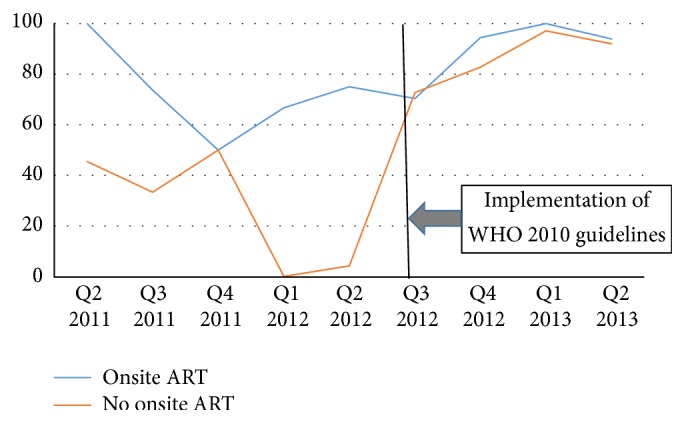
Proportions of HIV/TB coinfected patients initiated on ART by the end of TB treatment in clinics with and without onsite HIV services in Kisangani.

Overall, prior to the implementation of the WHO 2010 guidelines, whether in Kinshasa or in Kisangani, the proportion of HIV/TB patients referred for HIV care who were initiated on ART fluctuated from quarter to quarter. However, in general, a greater proportion of coinfected patients in clinics with onsite ART services were initiated on ART compared to patients in clinics without onsite ART services. After implementation of the WHO 2010 guidelines, the gap between clinics with and without onsite ART services narrowed substantially (Figures 1 and 2).

Specifically, in Kinshasa, of the 1,799 HIV/TB patients referred for HIV care, 633 had been referred before implementation of the new guidelines including 507 from clinics with onsite ART and 126 from clinics without onsite ART services. Of those, 401 (79.1%) and 63 (50%), respectively, were reported to have been initiated on ART. The PR comparing ART initiation in clinics with that of clinics without onsite ART services was 1.58 (95% CI 1.32, 1.89) (Table 2).

Similarly, in Kisangani, of the 418 HIV/TB patients referred for HIV care, 120 were before the implementation of the WHO 2010 guidelines including 76 from clinics with onsite ART and 44 from clinics without onsite ART services. Of those, 53 (69.7%) and 16 (36.4%), respectively, were reported to have been initiated on ART: PR 2.10 (95% CI 1.40, 3.16) (Table 2).

Following implementation of the 2010 guidelines, in Kinshasa, 1,166 HIV/TB patients including 795 and 371, respectively, from clinics with and without onsite ART services were referred for HIV care. Of those, 714 (89.8%) and 345 (93.0%), respectively, were initiated on ART. There was no statistical difference in the proportion of HIV/TB patients initiated on ART in TB clinics with and without onsite ART services after implementation of the 2010 guidelines in Kinshasa: PR = 0.97 (95% CI 0.93, 1.00).

In Kisangani, after implementation of the new guidelines, 298 HIV/TB patients including 152 and 146, respectively, from clinics with and without onsite ART service were referred for HIV care. Of those, 135 (88.8%) and 106 (72.6%), respectively, were initiated on ART. Despite having narrowed down substantially, compared to clinics without onsite ART services, ART initiation remained significantly higher in clinics with onsite services: PR = 1.22 (95% CI 1.09, 1.37) (Table 2).

Overall, implementation of the 2010 WHO guidelines resulted in significant increase in the proportion of TB/HIV patients initiated on ART whether in Kinshasa or in Kisangani. Comparing the proportions of coinfected patients initiated on ART during the period following implementation to the proportion of those initiated during the period before, in Kinshasa, the proportion ratios were 1.14 (95% CI 1.08, 1.19) in clinics with onsite ART services and 1.86 (95% CI 1.56, 2.22) in clinics without onsite ART services. In Kisangani, the proportion ratios were 1.27 (95% CI 1.09, 1.49) and 2.00 (95% CI 1.33, 2.99), respectively, in clinics with and without onsite ART services (Table 2).

## 4. Discussion

Our aim was to assess the impact of removing the need for CD4 count evaluation before ART initiation brought about by the WHO 2010 guidelines and that of HIV services decentralization on the proportion of HIV/TB patients initiated on ART before the end of TB treatment. Our results show that implementation of the 2010 WHO guidelines resulted in substantial and statistically significant increased proportions of HIV/TB patients who were initiated on ART. The increase was sufficient to erase the statistically significant difference in the proportions of HIV/TB initiated on ART in clinics with and without ART services in Kinshasa. However, in Kisangani, clinics without onsite ART services continued to perform significantly lower in their ART initiation coverage.

To the best of our knowledge, this is the first study evaluating the impact of the 2010 change in WHO guidelines on access to ART among HIV/TB patients. Our finding that removal of the need for a CD4 count before initiation ART substantially increased the proportion of HIV/TB patients that were initiated on ART supports the public health goal of those recommendations and may appear obvious. However, despite the substantial and significant increase, the proportions of HIV/TB patients initiated on ART remained below those initiated on CPT even in clinics with onsite ART services and far below the 100% target particularly in the more provincial town of Kisangani. This is consistent with previous observations of substantial delays in ART initiation even in clinics with integrated TB and HIV services due to the lack of implementation fidelity to treatment guidelines [14]. These delays are associated with increased mortality [15, 16] and while efforts to decentralize HIV services should continue, increased attention should be given to implementation fidelity of treatment guidelines. The lower proportion of patients initiated on ART compared to those initiated on CPT can be explained at least in part by the need for clinicians to wait for four to eight weeks to ensure that TB treatment is well tolerated before ART initiation, a restriction that does not apply to CPT.

Previous studies from South Africa showed that nonintegrated HIV and TB services negatively affect the timing of ART, mainly because of prolonged referral times in moving between TB and ART services [10, 11]. Our finding that implementing the 2010 WHO guidelines can result in decreasing clinical management differences among HIV/TB patients depending on onsite ART availability is welcome news and suggests that, with the “hub and spoke” strategy where very small clinics that might go for a year or longer without diagnosing a new HIV patient referred those cases to larger clinics, effective ART services can also be scaled up rapidly for HIV/TB coinfected patients.

The limitations of our study include the use of aggregated data. This prevented us from assessing the exact timing of ART initiation among patients. The proportion of patients initiated on ART should be interpreted as the proportion initiated before the end of TB treatment: six or eight months for new or retreatment cases. We do not know if all patients who were not referred were still in their first eight weeks of TB treatment. Moreover, we also lack information on why some patients who were referred for HIV services were never initiated on ART even in clinics with onsite services. However, it is unlikely that loss to follow-up played an important role given the high TB treatment success rate in the country [3]. Finally, the lack of control in clinics where the WHO 2010 guidelines were not implemented makes it impossible to know whether the observed increases in the proportion of HIV/TB patients initiated on ART can be attributed entirely to the implementation of the new guidelines alone, though the strengths of the effects plead for more than merely confounding factors.

In conclusion, implementation of the 2010 WHO guidelines that removed the need for CD4 counts before ART initiation resulted in substantial increases in the proportion of HIV/TB patients initiated on ART and narrowed the initiation gap between clinics with and without onsite ART services. However, ART initiation remained below that of CPT and below the 100% target, particularly in clinics without onsite ART services.

## Figures and Tables

**Table 1:** Numbers of TB cases counseled and tested for HIV by quarter and proportions of HIV+ cases initiated on cotrimoxazole prophylaxis and ART in Kisangani and Kinshasa.

Quarter	Number of clinics^a^	Total TB cases	With known HIV status *N* (%)	Number tested *N* (%)	HIV positive *N* (%)	Initiated on CXT *N* (%)	Total referred *N* (%)	Initiated on ART *N* (%)
	*Kinshasa*							
Q4 2010	20	844	Na	769 (91.1)	119 (15.5)	106 (89.1)	77	62 (80.5)
Q1 2011	20	1294	Na	1221 (94.4)	187 (15.3)	187 (100.0)	119	118 (99.2)
Q2 2011	20	1251	53 (4.2)	1150 (96.0)	109 (9.5)	162 (100.0)	90	77 (85.6)
Q3 2011	20	1359	57 (4.2)	1244 (95.5)	135 (10.9)	190 (99.0)	136	77 (56.6)
Q4 2011	32	1338	54 (4.0)	1164 (90.7)	111 (9.5)	161 (97.6)	82	62 (75.6)
Q1 2012	32	2054	73 (3.6)	1822 (92.0)	195 (10.7)	242 (90.3)	129	89 (69.0)
Q2 2012	32	2224	56 (2.5)	2067 (95.3)	206 (10.0)	260 (99.2)	167	141 (84.4)
Q3 2012	65	2361	35 (1.5)	2239 (96.3)	253 (11.3)	281 (97.6)	212	185 (87.3)
Q4 2012	65	2691	57 (2.1)	2596 (98.6)	269 (10.4)	325 (99.7)	233	216 (92.7)
Q1 2013	65	2776	54 (1.9)	2559 (94.0)	299 (11.7)	348 (98.6)	274	251 (91.6)
Q2 2013	65	2720	68 (2.5)	2518 (94.9)	298 (11.8)	363 (99.2)	280	268 (95.7)

*Total *	*65*	*20912*	*507 (2.5)*	*19349 (94.8)*	*2181 (11.3)*	*2625 (97.7)*	*1799*	*1546 (85.9)*

	*Kisangani*							
Q2 2011	3	189	13 (6.9)	149 (84.7)	33 (13.4)	44 (95.7)	22	16 (72.7)
Q3 2011	6	270	18 (6.7)	248 (98.4)	44 (10.5)	58 (93.5)	28	17 (60.7)
Q4 2011	6	252	24 (9.5)	207 (90.8)	53 (14.0)	53 (68.8)	32	16 (50.0)
Q1 2012	6	305	36 (11.8)	221 (82.2)	73 (16.7)	69 (63.3)	38	20 (52.6)
Q2 2012	6	327	33 (10.1)	229 (77.9)	65 (14.0)	63 (64.3)	44	16 (36.4)
Q3 2012	13	371	11 (3.0)	348 (96.7)	66 (15.8)	73 (94.8)	60	43 (58.9)
Q4 2012	13	406	20 (4.9)	385 (99.7)	74 (14.0)	93 (98.9)	65	59 (90.8)
Q1 2013	13	368	9 (2.4)	350 (97.5)	80 (20.3)	88 (98.9)	71	70 (98.6)
Q2 2013	13	500	11 (2.2)	489 (100.0)	78 (13.7)	89 (100.0)	58	54 (93.1)

*Total *	*13*	*2988*	*175 (5.9)*	*2626 (94.8)*	*566 (14.9)*	*630 (85.0)*	*418*	*311 (74.4)*

^a^Number of clinic contributing data.

**Table 2:** Effect of the WHO 2010 guidelines and that of decentralization of HIV services on ART initiation among HIV/TB patients in TB clinics in Kinshasa and Kisangani, Democratic Republic of Congo.

TB type	Population^b^	Period before^a^		PR (95% CI)^d^	Period after^a^		PR (95% CI)^d^	PR 95% CI^f^
		Eligible^c^	Initiated on ART		Eligible^c^	Initiated on ART		
Kinshasa	Onsite ART	507	401 (79.1)	1.58 (1.32, 1.89)	795	714 (89.8)	0.97 (0.93, 1.00)	1.14 (1.08, 1.19)
	No onsite ART	126	63 (50.0)	1	371	345 (93.0)	1	1.86 (1.56, 2.22)

Kisangani	Onsite ART	76	53 (69.7)	2.10 (1.40, 3.16)	152	135 (88.8)	1.22 (1.09, 1.37)	1.27 (1.09, 1.49)
	No onsite ART	44	16 (36.4)	1	146	106 (72.6)	1	2.00 (1.33, 2.99)

^a^Period before and after implementation of guidelines of WHO 2010: January 2012 in Kisangani and March 2012 in Kinshasa. ^b^Clinics with onsite (colocated) ART service or without.^c^HIV/TB patients referred for HIV care including ART. ^d^Log-binomial model estimates comparing the proportion of HIV/TB patients initiated on ART by type of clinic. ^f^Log-binomial model estimates comparing the proportion of HIV/TB patients initiated on ART by period after and before (after period = index). PR: proportion ratio. 95% CI: 95% confidence interval.
